# The complete mitochondrial genome of Asian short-toed Lark *Alaudala cheleensis* (Aves: Passeriformes: Alaudidae)

**DOI:** 10.1080/23802359.2021.1872447

**Published:** 2021-02-11

**Authors:** Ronghua Deng, Zhaohui Fu, Chao Du

**Affiliations:** Baotou Teachers College, Baotou, People’ s Republic of China

**Keywords:** Asian short-toed Lark, *Alaudala cheleensis*, mitochondrial genome phylogeny

## Abstract

We sequenced the mitochondrial genome of Asian short-toed Lark *Alaudala cheleensis* using the next-generation sequencing. The circular genome is 16,914 bp long, encoding 13 protein-coding genes (PCGs), 22 transfer RNAs (tRNAs), 2 ribosomal RNAs (rRNAs), and there are two control regions, which is similar to the common type suggested as ancestral for birds but has a 1126 bp control region and a 236-bp remnant control region. The phylogenetic analysis of published lark mitogenomes reveals the top phylogenetic position of *A. cheleensis* in Alaudidae.

The Asian short-toed Lark (*Alaudala cheleensis*) is a resident songbird in a large area of northeast and Western north China. The Asian short-toed Lark (*A. cheleensis*) and the Lesser short-toed Lark (*A. rufescens*) are a species complex in the genera *Alaudala*, and the Asian short-toed Lark has been considered to belong to the genus *Calandrella* or to be a subspecies of the Lesser short-toed Lark (Christian 2019; Ghorbani et al. 2020). A densely sampled phylogenetic analysis of larks based on 11 nuclear markers and the mitochondrial *cob* indicated that neither of these two larks is monophyletic (Ghorbani et al. 2020). Here, we sequenced and assembled the complete mitochondrial genome of *A. cheleensis*. The information would be helpful for taxonomic and phylogenetic studies of the family Alaudidae.

The sample was taken from a specimen in the museum of Baotou Teachers College (specimen No. BA840045), which was collected in the summer of 1984 at Sonid left banner, Xilingol League, Inner Mongolia, China (43°42′28.5″N, 114°09′56.1″E). The genomic DNA extraction, library construction, sequencing, assembly, and annotation were performed according to the methods described by Du et al. (2020a, 2020b). The annotated mitogenome was deposited in the GenBank database under accession no. MW143077.

The complete mitochondrial genome of *A. cheleensis* is a circular genome with 16,914 bp in length, which comprises 13 protein-coding genes (PCGs), 22 transfer RNAs (tRNAs), 2 ribosomal RNAs (rRNAs), one control region, and a remnant control region. Twelve PCGs and 14 tRNAs are encoded in the H-strand, apart from the *nad6* and the other eight tRNAs (*trnQ*, *trnA*, *trnN*, *trnC*, *trnY*, *trnS*, *trnP*, and *trnE*), as the ancestor type for avian mitogenome (Du et al. 2020a). The lengths of *rrnL* and *rrnS* were determined to be 1549 and 982 bp, respectively. The mitogenome has a 1126 bp control region and a 236 bp remnant control region. The nucleotide base composition of *A. cheleensis* mitogenome is 29.73% A, 33.63% C, 14.54% G, and 22.09% T, with an overall A + T content of 51.82%.

To infer the taxonomic status of *A. cheleensis*, we conducted the phylogenetic analysis of Alaudidae based on 13 mitochondrial PCGs. All five larks with published mitogenomes were used to perform the maximum likelihood analysis using IQ-TREE 2 (Minh et al. 2020). The resulting tree indicates that *A. cheleensis* possesses a top position in the family Alaudidae (Figure 1). Due to the limited sequence data of mitogenomes in the family, the taxonomic status of Asian/Lesser short-toed larks has not been clearly defined in this study. However, the complete mitogenome of *A. cheleensis* would be useful in the future to elaborate the taxonomy and phylogeny of this species complex.

**Figure 1.**
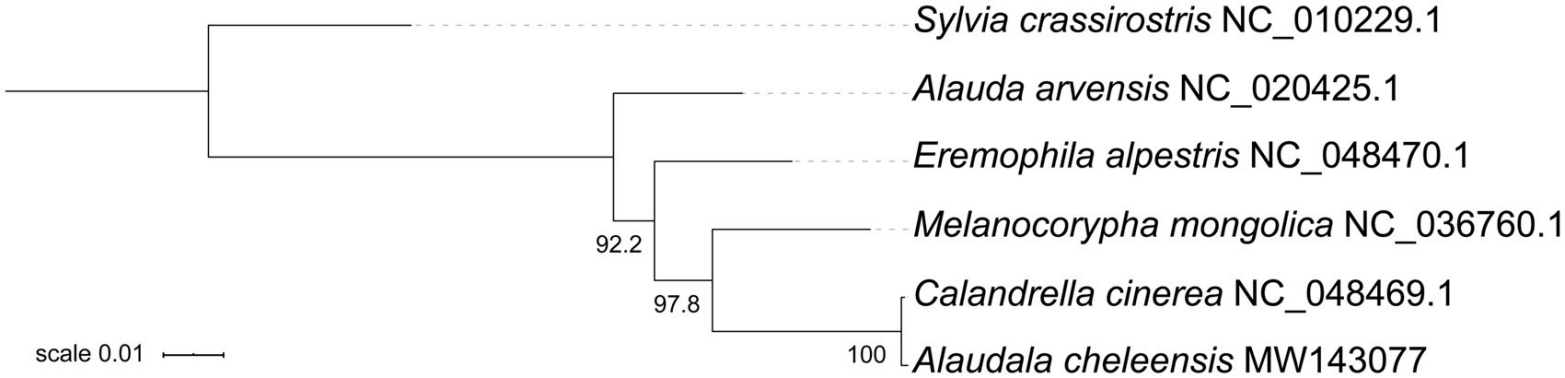
The maximum likelihood tree of Alaudidae based on the amino acid sequences of 13 PCGs, with *Sylvia crassirostris* as the outgroup. Support values are denoted next to nodes after 1000 bootstrap replicates.

## Data Availability

The genome sequence data that support the findings of this study are openly available in GenBank of NCBI at (https://www.ncbi.nlm.nih.gov/) under the accession no. MW143077. The associated BioProject, SRA, and Bio-Sample numbers are PRJNA685327, SRR13255425, and SAMN17082865 respectively.
